# Oxidative Stress Parameters and Morphological Changes in Japanese Medaka (*Oryzias latipes*) after Acute Exposure to OA-Group Toxins

**DOI:** 10.3390/life13010015

**Published:** 2022-12-21

**Authors:** Diego Figueroa, Javiera Ríos, Oscar F. Araneda, Héctor R. Contreras, Miguel L. Concha, Carlos García

**Affiliations:** 1 Laboratory of Marine Toxins, Physiology and Biophysics Program, Institute of Biomedical Sciences, Faculty of Medicine, Universidad de Chile, Santiago 8380000, Chile; 2 Integrative Biology Program, Institute of Biomedical Sciences, Faculty of Medicine, Universidad de Chile, Santiago 8380000, Chile; 3 Integrative Laboratory of Biomechanics and Physiology of Effort, Kinesiology School, Faculty of Medicine, Universidad De Los Andes, Santiago 7620086, Chile; 4 Laboratory of Cellular and Molecular Oncology, Department of Basic and Clinical Oncology, Faculty of Medicine, University de Chile, Santiago 8380453, Chile

**Keywords:** *Oryzias latipes*, okadaic acid, dinophysistoxin, oxidative stress

## Abstract

Toxins of the OA-group (okadaic acid, OA; dinophysistoxin-1, DTX-1) are the most prevalent in the fjords of southern Chile, and are characterized by their potential harmful effects on aquatic organisms. The present study was carried out to determine the acute toxicity of OA/DTX-1 on oxidative stress parameters in medaka (*Oryzias latipes*) larvae. Medaka larvae were exposed to different concentrations (1.0–30 μg/mL) of OA/DTX-1 for 96 h to determine the median lethal concentration. The LC_50_ value after 96 h was 23.5 μg/mL for OA and 16.3 μg/mL for DTX-1 (95% confidence interval, CI was 22.56, 24.43 for OA and 15.42, 17.17 for DTX-1). Subsequently, larvae at 121 hpf were exposed to acute doses (10, 15 and 20 μg/mL OA and 5.0, 7.5 and 11.0 μg/mL DTX-1) for 96 h and every 6 h the corresponding group of larvae was euthanized in order to measure the activity levels of biochemical biomarkers (superoxide dismutase, SOD; catalase, CAT; glutathione peroxidase, GPx; and glutathione reductase, GR) as well as the levels of oxidative damage (malondialdehyde, MDA; and carbonyl content). Our results showed that acute doses caused a decrease in SOD (≈25%), CAT (≈55%), and GPx and GR (≈35%) activities, while MDA levels and carbonyl content increased significantly at the same OA/DTX-1 concentrations. This study shows that acute exposure to OA-group toxins tends to simultaneously alter the oxidative parameters that induce sustained morphological damage in medaka larvae. DTX-1 stands out as producing greater inhibition of the antioxidant system, leading to increased oxidative damage in medaka larvae. Considering that DTX-1 is the most prevalent HAB toxin in southern Chile, these findings raise the possibility of an important environmental impact on the larval stages of different fish species present in the southern fjords of the South Pacific.

## 1. Introduction

In the last 30 years, harmful algal blooms (HABs) have become a serious environmental problem, as their occurrence, distribution, magnitude, and persistence have increased worldwide. These components are mainly related to increased aquaculture, agricultural run-off, and global warming [1,2]. HABs are related to the production of toxins with different chemical and toxicological characteristics, which in the aquatic environment can affect and alter marine ecosystems [3,4,5].

The okadaic acid group (OA-group) consists of a polyether with fatty acids, which is produced by dinoflagellates of two genera, *Dinophysis* and *Prorocentrum* [6]. The most dominant analogues described in this group are okadaic acid (OA), dinophysistoxin-1 (DTX-1) and dinophysistoxin-2 (DTX-2) [7]. The toxin content and ratio of analogues produced by dinoflagellates tend to be species-specific and are associated with the region in which the blooms occur, such as those associated with *Dinophysis acuta* (n.d. ≈ 40 pg cell-1 OA/n.d. ≈ 0.02 pg cell-1 DTX-1) and *Dinophysis acuminata* (n.d. ≈ 160 pg cell-1 OA/n.d. ≈ 7.8 pg cell-1 DTX-1/n.d. ≈ 169 pg cell-1 DTX-2) [6,8]. However, microalgal density and thus toxin concentrations in the aqueous medium are linked to the interaction with abiotic/biotic factors that maintain or inhibit the bloom of toxin-producing species. Additionally, it should be considered that the release of toxins into the aquatic environment does not occur actively, and is therefore more associated with the senescent phases of the blooms; it is estimated that concentrations in the aqueous medium can reach a range of 0.4–0.8 μg/mL [6,9].

Constant HABs favor high densities of these primary producers and, at the same time, toxins in the aquatic environment, which are accumulated mainly by filter-feeding organisms [10]. The accumulation of OA-group toxins in mollusks and shellfish suggests that there is a possibility that these toxins can be biotransformed by acylation that modify the toxicity level of OA-group toxin analogues [11]. Thus, transfer through the food chain involves the assimilation of toxic analogues other than those produced by microalgae [12,13].

OA-group toxins are characterized by inhibiting serine/threonine protein phosphatases 1 (PP1) and 2A (PP2A) [14,15,16]. These inhibitions produce severe effects on intracellular processes such as metabolism, contractibility, inflammatory response, and maintenance of the cytoskeletal structure [17,18,19]. In addition, it is noteworthy that this group has been classified as a potent tumor promoter [20,21].

Marine organisms of the upper trophic level, such as fish, can accumulate OA-group toxins in their tissues. However, passive interaction conditions, such as the process of dilution and dispersion of toxins in the aquatic environment, in addition to the high mobility of fish, can result in very low levels of accumulation of OA-group toxins [6,22]. However, few data are available on the effects of exposure and biochemical responses in the early developmental stages of the larvae of fish.

Aquatic organisms, including fish, have developed a physiological antioxidant system to reduce damage from exposure to oxidative stress-producing xenobiotics, involving antioxidant enzymes, such as superoxide dismutase (SOD), catalase (CAT), glutathione peroxidase (GPx) and glutathione reductase (GR) [23]. Therefore, antioxidant enzyme activities are a suitable way to detect responses in fish against exposure to different toxin concentrations [24,25,26].

Experimentally, it has been shown that OA can cause oxidative stress in aquatic species such as bivalves through increased reactive oxygen species (ROS) and lipoperoxidation [26,27]. An excess of ROS can in turn cause intracellular damage that not only affects membrane lipids, but also proteins and DNA [28,29]. However, the alteration of antioxidant enzyme activity will depend mainly on the concentration and route of exposure to the xenobiotic or toxins involved [30].

Medaka (*Oryzias latipes*) is an oviparous fish that can tolerate wide ranges of salinity and temperature. It is easy to maintain and reproduce under laboratory conditions [31]. Its fertilization and embryogenesis are external, which represents one of the most important advantages as a model of study in developmental biology. The extensive genomic and physiologic characterization and availability of transgenic and mutant lines highlight it as a useful model for the study of different areas of biology, biomedicine and environmental sciences [32,33]. The use of medaka larvae as a study model allows determining multiple alterations on antioxidative enzymes during early ontogenesis, a stage in which they are more sensitive to environmental stressors. This allows us to more accurately establish the susceptibility levels of the whole organism when exposed to different concentrations of xenobiotics [34,35].

In this contribution, we tested the hypothesis that the toxins of the OA group produced by harmful algal blooms cause in medaka larvae a decrease in the activity of the enzymes of the antioxidant defense system, which favors the oxidative damage and morphological changes. The objective of this research was to determine the toxicity and biochemical response of the antioxidant defense system to acute exposure to OA and DTX-1, using medaka larvae as a model.

## 2. Materials and Methods

### 2.1. Materials

Okadaic acid (OA) (M.W. 827; CAS: 209266-80-8; 100 mg) and dinophysistoxin-1 (DTX-1) (M.W. 819; CAS: 81720-10-7; 100 mg) were purchased from Abcam (Cambridge, UK) and Cayman Chemicals (Ann Arbor, MI, USA) respectively. Toxins were resuspended in 100 mM dimethyl sulfoxide (DMSO). Commercial assay kits of SOD, CAT, GPx, and GR were purchased from Sigma-Aldrich (Sigma-Aldrich; Merck KGaA, Darmstadt, Germany). All other reagents were of analytical grade.

### 2.2. Fish Husbandry and Embryo Collection

The experiments and procedures in this project were approved by the Faculty of Medicine, Universidad de Chile, Bioethical Committee (CBA 0862 FMUCH) and the Institutional Biosafety Committee project (Project No 1160168).

The wild-type Cab strain of Japanese Medaka (*Oryzias latipes*) was obtained from the fish facility of the Laboratory of Experimental Ontogeny, Institute of Biomedical Sciences, Faculty of Medicine at University of Chile. Adult fish were maintained in a multi-tank, recirculating water system with a 14:10 daily light:dark cycle at 28 ± 1 °C. Embryos were obtained by natural spawning, raised at 26–28.5 °C in embryo-rearing medium (ERM) (17 mM NaCl, 0.4 mM KCl, 0.6 mM MgSO_4_, 0.36 mM CaCl_2_, with a conductivity at 340 ± 10 μS, pH adjusted to 7.4 ± 0.3 with NaHCO_3_ and 0.0002% methylene blue to reduce fungal infection), and staged according to morphology [36] and age in hours or days post fertilization (hpf and dpf, respectively). Older embryos were mechanically dechorionated; the process was carried out by means of two pairs of forceps or dissection needles. However, the mechanical stress frequently resulted in destruction of the embryo. For younger embryos, enzymatic dechorionation was performed using pronase and hatching enzyme. The embryos were placed in a small drop of water on a plastic surface, excess liquid was removed and hatching enzyme was added (10 μL hatching enzyme per 15 embryos). The embryos were incubated at 27 °C until holes appeared in the chorion. The remaining chorion was removed using forceps and the embryos were kept in ERM in dishes coated with agarose. Hatching enzyme was prepared as follows: embryos with visible hatching glands were homogenized in pre-cooled PBS (0.75 μL PBS per embryo), incubated overnight at 4 °C and debris was removed by centrifugation (15,000 rpm, 4 °C, 10 min). The hatching enzyme solution was stored in aliquots at −20 °C [37,38].

Normal stages of development were determined according to the morphologic event described by this model and their chronological age (hpf) [36,39]. Subsequently, after examining the larvae and establishing the morphological alterations produced by the assay, the larvae were recorded as either normal or abnormal (presence of one or more morphological abnormalities) [26].

### 2.3. Median Lethal Concentration (LC_50_)

A group of medaka larvae was selected and used to determine the median lethal concentration (LC_50_) at 96 h of exposure. Larvae at 60 hpf were placed in 24-well cell culture plates, and were arranged in an incubator with photoperiodic lighting (14:10 daily light:dark cycle at 28.5 °C). Each well contained five medaka larvae with a volume of 900 μL of ERM solution (17 mM NaCl; 0.4 mM KCl; 0.6 mM MgSO_4_; 0.36 mM CaCl_2_; pH adjusted to 7.4 with NaHCO_3_ and 0.0002% methylene blue to reduce fungal infection) and 100 μL of OA-group toxin solution at different concentrations (1.0; 5.0; 10.0; 15.0; 16.0; 20.0; 23.5; 27.0 and 30 μg/mL for OA, and 1.0; 4.0; 7.0; 11.0; 16.0; 21.0; 25.0; 27.0 and 30.0 μg/mL for DTX-1). All OA-group toxin concentrations included their corresponding control groups (0 μg/mL); the ERM solution contained the same amount of solvent used in OA group toxins. Data are presented as mean ± SEM of at least three separate experiments. Each experiment was performed in triplicate.

### 2.4. Morphological Abnormalities

The median effective concentration (EC_50_) was established using the response of larvae to different concentrations of the toxins used (<LC_50_). Larvae at 60 hpf were placed in 24-well cell culture plates, and were arranged in an incubator with photoperiodic lighting (14:10 daily light:dark cycle at 28.5 °C). Each well contained five medaka larvae with a volume of 900 μL of ERM solution (17 mM NaCl; 0.4 mM KCl; 0.6 mM MgSO_4_; 0.36 mM CaCl_2_; pH adjusted to 7.4 with NaHCO_3_ and 0.0002% methylene blue to reduce fungal infection) and 100 μL of OA-group toxin solution at concentrations of 5.0; 10.0; 15.0; 16.0 and 20.0 for OA, and 4.0; 5.0; 7.5; 11.0 and 15.0 μg/mL for DTX-1. Each of the OA-group toxin concentrations was compared with its respective control group. Each experiment was performed in triplicate with its respective control to evaluate reproducibility.

All larvae exposed to OA group toxins were observed three times a day over a 96 h period. The responses to determine EC_50_ in larvae exposed to different concentrations corresponded to edema, cyclopia, shortening of the anterior–posterior axis, developmental delay, lethargy, paresis, and erratic swimming [26,40,41]. The sizes were determined by measuring the length of the larvae exposed to different concentrations of OA-group toxins and they were compared with their respective controls by using a stereomicroscope (SMZ1270, Nikon, Nikon Instruments Inc., Melville, NY, USA) equipped with a calibrated ocular micrometer. Observations were performed at room temperature [42]. To detect the immobility of the larvae by exposure to OA-group, a tactile stimulus in the tail was used. This stimulus causes healthy larvae to immediately move (swim) away from the source of the stimulus. This process was found with larvae in a normal state [26]. Larvae that generally did not have any mobility were directly associated with morphological abnormalities, which was rigorously recorded by microscopic observation and tabulated according to the time and concentration used to determine EC_50_ [36,39]. Immobilized larvae were euthanized with 0.02% tricaine methanesulfonate (MS-222) [35,43].

### 2.5. Calculation of the Median Lethal and Median Effective Concentrations

The calculation of 96 h LC_50_ was obtained by plotting the percentage of mortality versus the concentration of OA-group toxins using Probit analysis. The variation of concentrations of OA-group toxins of the analyzed samples was performed using a t-test and the relationship between the exposure concentration and the percentage of mortality was performed using linear regression analysis [44,45].

For OA and DTX-1, the EC_50_ values and concentration–response curves were calculated using a non-linear regression analysis with a four-parameter logistic (4PL) equation in the GraphPad PRISM software, version 4.0. [46].

### 2.6. Evaluating Time Course of Adverse Effects in Older Larvae

According to data obtained from the EC_50_ test with younger larvae, a group of medaka larvae at 121 hpf was selected and incubated at different concentrations of OA-group toxins in order to estimate their growth rates, morphological abnormalities, biochemical biomarkers and oxidative damage.

Each well contained five medaka larvae (121 hpf) with a volume of 900 μL of ERM solution and 100 μL of OA-group toxin solution at different concentrations (10.0; 15.0 and 20 μg/mL for OA, and 5.0; 7.5 and 11.0 μg/mL for DTX-1). The control solution consisted of ERM solution containing the same amount of solvent agent used in OA group toxins. Larvae were incubated in 24-well cell culture plates (14:10 daily light:dark cycle at 28.5 °C). After 6 h, 12 h, 24 h, 48 h, 72 h and 96 h of exposure, larvae from each concentration were euthanized and used to determine the alterations of biochemical biomarkers and oxidative damage produced by OA and DTX-1. Each experiment was performed in triplicate for each analogue to evaluate reproducibility [26,41].

### 2.7. Determination of Biochemical Biomarkers

The medaka larvae group exposed to the different concentrations of OA-group toxins was collected and ultrasonically lysed on ice in a TRIS buffer (100 mM, pH 7.8; 1:10 *w*/*v*) using an Ultra-Turrax^®^ (D25 basic, IKA©-Werke, Staufen, Germany). After centrifuging at 21,000× *g* for 5 min at 4 °C, the resulting supernatant was diluted (10% *v*/*v*) and used to determine the activities of the enzymes SOD, CAT, GPx and GR [26,47]. The enzymatic parameters were analyzed in triplicate using appropriate reagents and detection kits (Sigma Aldrich, St. Louis, MO, USA).

To determine superoxide dismutase (SOD) activity, we followed the method described by Simos et al. [48]. Along with 50 μL of freshly prepared supernatant obtained from the lysate of medaka larvae, 1.3 mL of WST-1 ([2-(4-iodophenyl)-3-(4-nitrophenyl)-5-(2,4-disulfophenyl)-2H-tetrazolium, monosodium salt]) was incubated. The reaction was initiated by the addition of 10 μL xanthine oxidase (50 μM), and incubated for 20 min at 37 °C, and then the absorbance at 450 nm was determined (Biotek Synergy HT Multi-mode microplate reader, BioTek, Highland Park, Winooski, VT, USA). The SOD activity was defined as the amount of inhibitor required to reduce the activity of 1 mL solution by 50% and was expressed as units U/mg total proteins (U/mg).

To determine catalase (CAT) activity, we followed the method described by Kowaltowski et al. [49]. Supernatant of the medaka larvae lysate (50 μL) was supplemented with 1.1 mL of 500 mM potassium phosphate buffer (pH 7.0) at room temperature, and then 20 μL of H_2_O_2_ (20 mM) was added. The reaction was mixed and incubated for 5 min at room temperature. Then, the reaction was stopped with the addition of 15 μL of sodium azide (15 mM). From the final volume, 10 μL was taken and mixed with 1.0 mL of potassium phosphate buffer (150 mM), pH 7, containing 0.25 mM 4-aminoantipyrine and 2 mM 3.5-dichloro-2-hydroxybenzenesulfonic acid, and then incubated for 15 min at room temperature. The final reaction was analyzed at 520 nm absorbance (Biotek Synergy HT Multi-mode microplate reader). CAT activity was defined as the amount of 1 μmol of H_2_O_2_ metabolised/mg proteins (U/mg).

To determine glutathione reductase (GR) activity, we followed the method described by Han and Han [50]. GR activity was measured by incubating 500 μL 2 mM of oxidized glutathione in 350 μL 100 mM potassium phosphate buffer, with 1 mM EDTA and 0.1 mM KCl (pH 7.5). Then, 30 μL freshly prepared supernatants from the medaka larvae was added followed by 50 μL NADPH (2 mM). After 5 min preincubation (37 °C), the reaction was initiated by the addition of 100 μL GSSG (1 mM). The final reaction volume was analyzed at 340 nm absorbance (Biotek Synergy HT Multi-mode microplate reader). One unit of GR was defined as the amount of enzyme that will reduce 1 μmol of glutathione (GSSG)/min.

To determine glutathione peroxidase (GPx) activity, we followed the method described by Gupta and Baquer [51]. GPx activity was determined by incubating 930 μL Tris HCl (50 mM), containing 0.5 mM EDTA (pH 8); 50 μL 5 mM NADPH, 50 μL 42 mM reduced glutathione, 10 units/mL of glutathione reductase, and 50 μL supernatants from the medaka larvae. After 5 min of preincubation at room temperature, the reaction was initiated by the addition of 10 μL 30 mM tert-butyl-hydroperoxide. The final reaction was analyzed at 340 nm absorbance (Biotek Synergy HT Multi-mode microplate reader). One unit of GPx activity corresponded to a decrease of 1 μmol GSH/mg protein/min (U/mg).

### 2.8. Oxidative Damage

To determine the carbonyl content, we followed the method described by Parraguez et al. [52]. The supernatant of medaka larvae was centrifuged at 21,000× *g* for 5 min at 4 °C and the resulting volume was incubated for 15 min with 10% streptomycin sulphate at 4 °C to eliminate DNA debris. From the resulting mixture, 100 μL of supernatant and 100 μL of fresh 10 mM 2,4-dinitrophenylhydrazine (DNPH) prepared in 2M HCl was added. The contents were mixed and incubated in the dark at room temperature for 10 min. After the time had elapsed, 30 μL of 100% trichloroacetic acid (TCA) (*w*/*v*) was added and the tube was incubated for 15 min on ice, to be then centrifuged at 7200× *g* for 15 min at 4 °C to collect the protein pellet. The supernatant was aspirated and discarded. The precipitates were dissolved in 200 μL 6 M guanidine hydrochloride and incubated at 37 °C for 10 min. The insoluble materials were removed by centrifugation (11,000× *g* for 3 min at 4 °C) and 100 μL was used to determine the content of carbonyl at 375 nm absorbance. Results were expressed as nmol carbonyl per mg proteins.

To determine the lipid peroxidation (LPO) content, we followed the method described by Zeb and Ullah [53]. The tissue of medaka larvae (0.5 mL) was homogenized in an ice bath with 30 μL of butylated hydroxytoluene (BHT) 1% mass/vol in glacial acetic. Then, the samples were centrifuged at 21,000× *g* for 10 min at 4 °C. From the supernatant, a sample of 200 μL was used and 600 μL thiobarbituric acid (TBA, 1%) was added. The mixture was then heated at 95 °C for 60 min. Subsequently, the reaction was cooled in an ice bath for 10 min. Of this reaction, 200 μL was used to determine the content of TBA at 532 nm absorbance. TBARS concentrations were derived from an external standard curve of 1,1,3,3-tetramethoxypropane (malondialdehyde; MDA). Peroxidized lipid content was calculated as nmol TBA reactive substance (nmol TBARS)/mg protein.

### 2.9. Protein Concentration

Protein concentration was determined using the Bradford method (Bio-Rad, Proteinassay, Hercules, CA, USA) [54]. The standard curve was generated using bovine serum albumin (BSA) as standard.

### 2.10. Mass Spectrometer Coupled to High-Resolution Liquid Chromatography

For OA and DTX-1 analysis, an ion trap mass spectrometer coupled to an Agilent 1200 Series LC system (Esquire 4000 ESI-IT, Bruker Daltonik GmbH, Billerica, MA, USA) was used. The Purospher STAR C-18 HPLC column, 50 × 2.1 mm, with a particle size of 3 μm (Hibar HR Purospher STAR, Merck KGaA 64271, Darmstadt, Germany) was used to separate the analytes. The elution flow consisted of an isocratic phase of 40% *v*/*v* mobile phase A (100% water with 2 mM ammonium formate/50 mM formic acid) and 60% *v*/*v* mobile phase B (95% acetonitrile with 2 mM ammonium formate/50 mM formic acid). The mass spectrometer was used for multiple reactions, using nitrogen as the collision gas. Nebulization ionization was performed at 4000 V, at a temperature of 300 °C, a pressure of 30 psi and a flow of 10 L min^−1^. Chromatograms and mass spectra were obtained according to the following protocol: a time of 3.1 min for OA; MRM, *m*/*z* 803 with the transitions of *m*/*z* 803→254.9, *m*/*z* 803→563 and *m*/*z* 803→765 and 817; a time of 5.0 min for DTX-1 with the transitions: *m*/*z* 817→254.9 and *m*/*z* 817→563. Negative polarities of both analytes were evaluated [11,55].

### 2.11. Statistical Analysis

Results are expressed as mean ± SEM (*n* = 3). Calibration curves were obtained through regression analyses. Differences between groups were analyzed using a one- or two-way analysis of variance (ANOVA) depending upon the number of variables to analyze. Previously, the normality of the distributions was evaluated with the Shapiro–Wilk Test while the homogeneity of variance was determined with the Levene test. A *p* < 0.05 significance level was considered for all cases. Analyses were performed using GraphPad Prism software (GraphPad Prism 7, GraphPad Software Inc., La Jolla, CA, USA).

## 3. Results

### 3.1. Analysis of OA-Group Toxins

**Figure 1:**
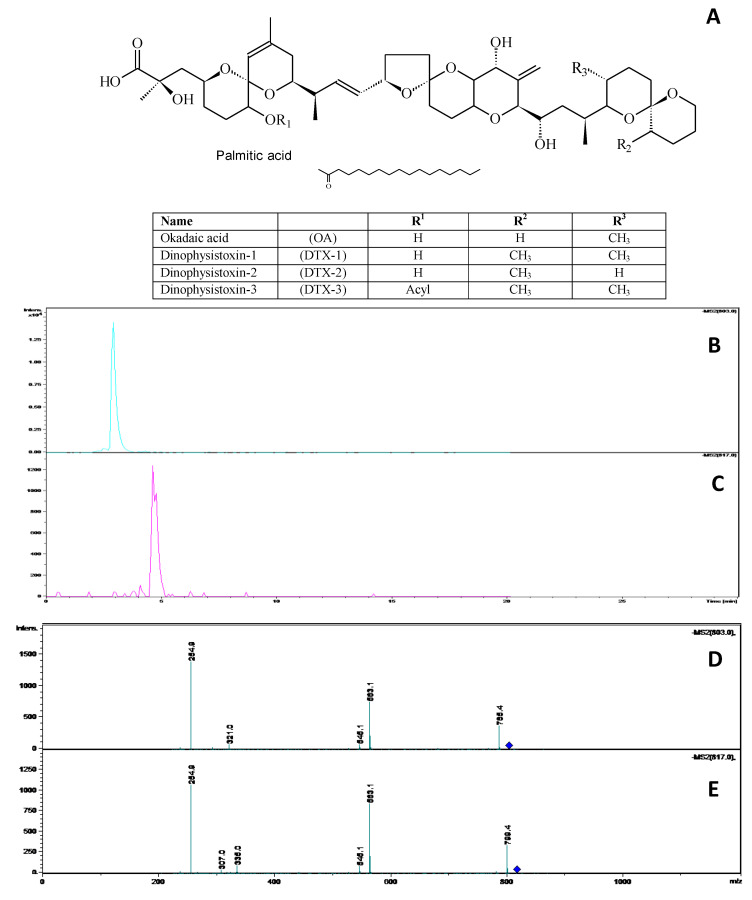
General structure of the okadaic acid group. Palmitic acid corresponds to the most prevalent fatty acid in the C-7 acylation of the OA-group toxins (**A**); Liquid chromatography and MS/MS spectra analysis of Okadaic acid (OA) (**B**,**D**), and Dinophysistoxin-1 (DTX-1) (**C**,**E**).

Detection and quantification of OA-group toxin analogues (OA and DTX-1, Figure 1A) was carried out using LC-MS/MS according to the observation of analyte signals, showing molecular mass, retention time (R_t_), and fragmentation ions (Figure 1B–E). Standard solutions containing the verified concentration of OA or DTX-1 were diluted in 100 mM DMSO, and then prepared in ERM solution for exposure of medaka larvae in the range from 1.0 to 30 μg/mL (R^2^ = 0.9983). Regarding OA analysis, an R_t_ of 3:01 min was detected (Figure 1B), in which the presence of a molecular ion was identified at 803 *m*/*z* [M-H]^−^, with characteristic fragments corresponding to 254.5, 563.1 and 765 *m*/*z* (Figure 1D). Meanwhile, for DTX-1, a 5:00 min R_t_ was detected with a molecular ion corresponding to 817 *m*/*z* [M-H]^−^, with characteristic fragments at 254.9 and 563 *m*/*z* (Figure 1C,E). No analogues associated with toxin degradation or conversion were detected after 96 h incubation of OA-group toxins in ERM solution.

### 3.2. Effects of Okadaic Acid and Dinophysistoxin-1 on Medaka Larvae

**Figure 2:**
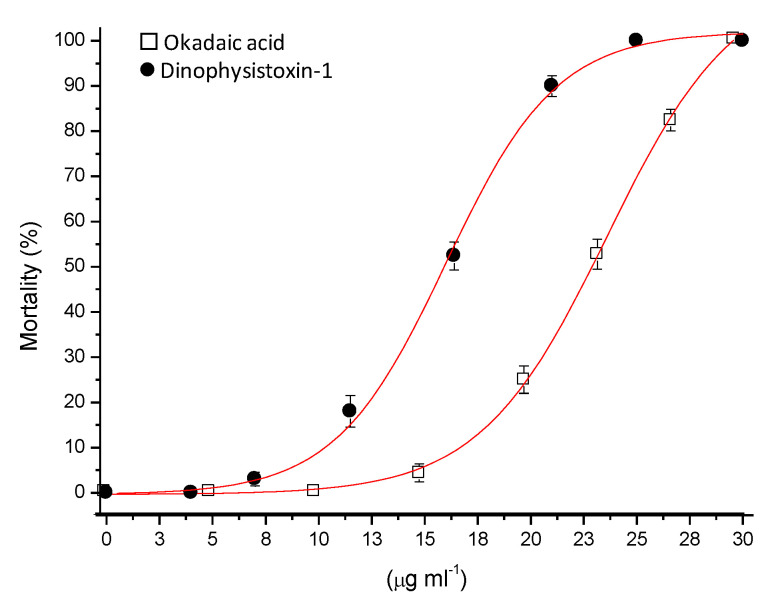
Mortality curve associated with exposure to OA-group toxins (OA and DTX-1). Concentrations are indicated as micrograms/mL (μg/mL) OA and DTX-1.

Exposure of medaka larvae to different concentrations of OA group toxins produced a decreased survival rate. Concentrations of 30 μg/mL of the OA group toxins produced a 100% decrease in the survival rate (112 hpf) relative to control exposure. This factor decreased to ≈80% as toxins were used at lower concentrations (27.5 μg/mL in OA or ≈20 μg/mL DTX-1). At concentrations ≤ 10 μg/mL OA or DTX-1, a decrease in survival rate was detected between 0–5% (Figure 2). The median lethal concentration (LC_50_) was estimated to be 23.5 μg/mL for OA, and 16.3 μg/mL for DTX-1 (95% confidence interval, CI 22.5 ± 1.0, 24.4 ± 1.3 for OA and 15.4 ± 1.5, 17.2 ± 1.2 for DTX-1, *n* = 3). In addition, a number of morphological anomalies was observed in medaka larvae after exposure to OA-group toxins (<LC_50_) (Figure S1), including severe pericardial edema, shortening of the anterior–posterior axis and developmental delay (Table 1).

### 3.3. Effects of OA-Group Toxins on the Growth and Development of Older Larvae

**Figure 3:**
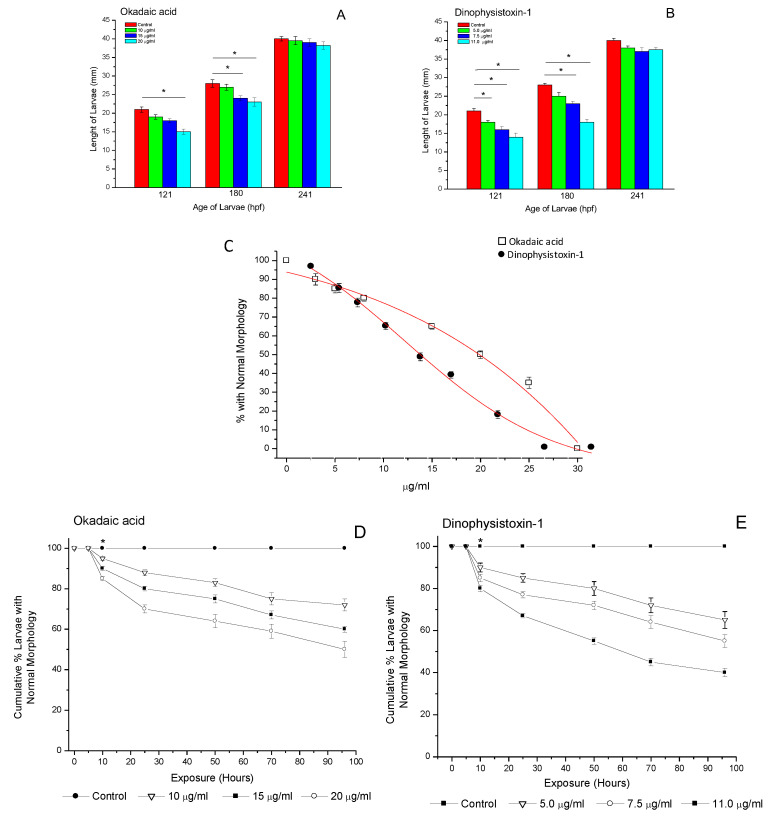
Growth rates of medaka larvae at 121, 180 and 241 hpf after being exposed to okadaic acid at 10; 15 and 20 μg/mL and dinophysistoxins-1 at 5.0; 7.5 or 11.0 μg/mL (**A**,**B**); EC_50_ values calculated at 96 h of exposure for medaka larvae exposed to various concentrations OA-group toxins (**C**); cumulative % larvae with normal morphology observed during 96 h exposures to okadaic acid and dinophysistoxin-1 (**D**,**E**). Each of the OA-group toxin concentrations was compared with its respective control group. All values are shown as mean ± SEM (*n* = 3) * Significant at *p* < 0.05.

Evaluation of growth and development was carried out on medaka larvae at 121, 180 and 241 hpf during exposure of OA-group toxins at concentrations of 10; 15 and 20 μg/mL for OA, and 5.0; 7.5 and 11.0 μg/mL for DTX-1. For larvae incubated at 121 hpf in OA at concentrations of 10, 15 and 20 μg/mL, morphological variability was observed, including a decrease in larval length by ≈25%. A similar tendency was observed in larvae incubated at 180 hpf (20%) while no significant changes were observed in larvae incubated at 241 hpf (Figure 3A). Similar results were observed for the incubation in DTX-1. Larvae incubated at 121 and 180 hpf showed a variability in length of 35% at concentrations of 5.0, 7.5 and 11.0 μg/mL (Figure 3B) while no significant changes were detected in larvae incubated at 241 hpf at the same concentrations. The median effective concentration (EC_50_) was estimated to be 20 μg/mL for OA, and 11 μg/mL for DTX-1 (95% confidence interval, CI 20.6, 19.4 for OA and 11.7, 10.3 for DTX-1) (Figure 3C). In addition, incubation at concentrations ≤ EC_50_ in OA and DTX-1 also caused a slight increase in anatomical abnormalities in medaka larvae, such as dorsal body curvature and edema, which were more evident in at 121 and 180 hpf larvae incubated in OA and DTX-1 (Figure S2).

Incubation of 121 hpf medaka larvae at concentrations ≤ EC_50_ of OA (10; 15 and 20 μg/mL) and DTX-1 (5.0; 7.5 and 11.0 μg/mL) showed variations in the percentage of larvae with normal morphology in relation to exposure time. Larvae incubated in OA showed a significant change in normal morphology at concentrations of 20 μg/mL from ≈10 h of incubation (≈25%, *p* < 0.05), which increased gradually between 25–96 h of incubation (≈40%, *p* < 0.05) (Figure 3D). At lower concentrations of OA (10 and 15 μg/mL), changes were also evident and significant from 10 h of incubation at concentrations of 15 μg/mL (≈20%, *p* < 0.05) and at 25 h of incubation at concentrations of 10 μg/mL (≈25%, *p* < 0.05). Similar results were obtained in the incubation of medaka larvae in DTX-1. The 11.0 μg/mL concentration showed significant changes in the normal morphology of medaka larvae at 10 h of incubation (≈13%, *p* < 0.05), which gradually increased between 25–96 h of incubation, with the maximum variability of alterations reached at 96 h (≈60%, *p* < 0.05) (Figure 3E). At lower concentrations of DTX-1 (5.0 and 7.5 μg/mL), changes were also observed at the same incubation times (variation of 50–28%, respectively, *p* < 0.05). No changes were observed in larvae exposed to control medium.

### 3.4. Temporal Evaluation of Incubation with OA-Group Toxins on Antioxidant Enzymes

**Figure 4:**
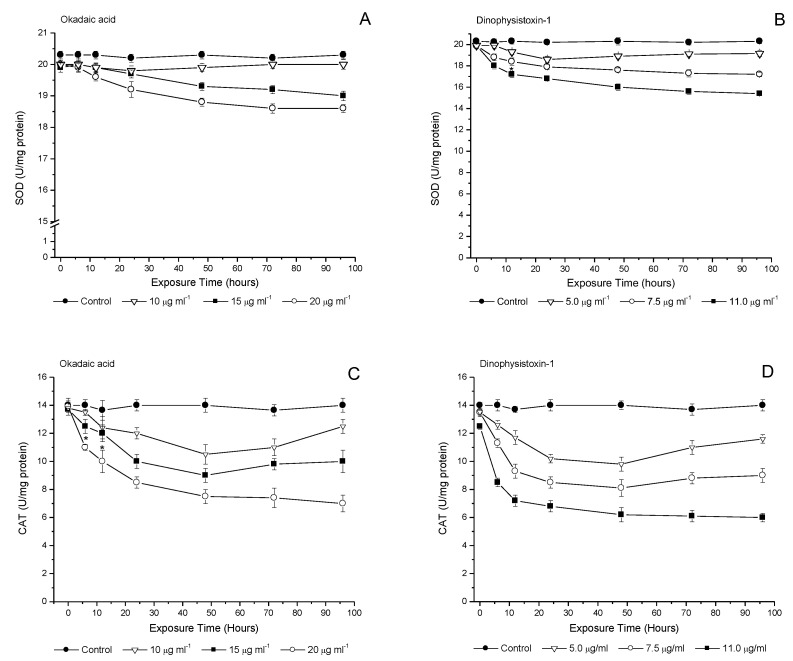
Superoxide dismutase (SOD) (**A**,**B**) and catalase (CAT) (**C**,**D**) activities in medaka larvae after exposure to okadaic acid (10; 15 or 20 μg/mL), and dinophysistoxins-1 (5.0; 7.5 or 11.0 μg/mL). Each of the OA-group toxin concentrations was compared with its respective control group. All values are shown as mean ± SEM (*n* = 3).

Antioxidant enzymes levels were analyzed in 121 hpf medaka larvae incubated for 96 h at concentrations of 10, 15 and 20 μg/mL for OA, and 5.0, 7.5 and 11.0 μg/mL for DTX-1 (Figure 4A–D). SOD levels in medaka larvae incubated with OA showed significant changes at concentrations of 15 and 20 μg/mL from 48 h and 24 h, respectively (*p* < 0.05), while at concentrations of 10 μg/mL, no changes in activity were found relative to the control incubation (Figure 4A). SOD levels upon incubation of medaka larvae with DTX-1 showed a significant decrease from 6 h of incubation at 7.5 and 11.0 μg/mL, and from 12 h of incubation at a concentration of 5.0 μg/mL (*p* < 0.05). The highest levels of decreased enzyme activity were detected between 48 and 96 h at concentrations of 7.5 and 11.0 μg/mL of DTX-1 (*p* < 0.05) (Figure 4B). The results on incubation with DTX-1 show that SOD inhibition is dependent on the concentration of the toxin.

CAT activity showed a decrease when compared to controls from 12 h OA incubation at a concentration of 10 μg/mL (≈25%, *p* < 0.05), while significant changes started at 6 h of incubation at concentrations of 15 and 20 μg/mL (≈20%, *p* < 0.05). At a concentration of 10 μg/mL with OA, CAT levels tended to increase from 72 h of incubation (Figure 4C). On the other hand, medaka larvae exposed to DTX-1 showed a more pronounced decrease in CAT activity with significant changes starting at 6 h of incubation at concentrations of 5.0, 7.5 and 11.0 μg/mL with DTX-1 (≈10%, 15% and ≈40%, respectively, *p* < 0.05) (Figure 4D).

**Figure 5:**
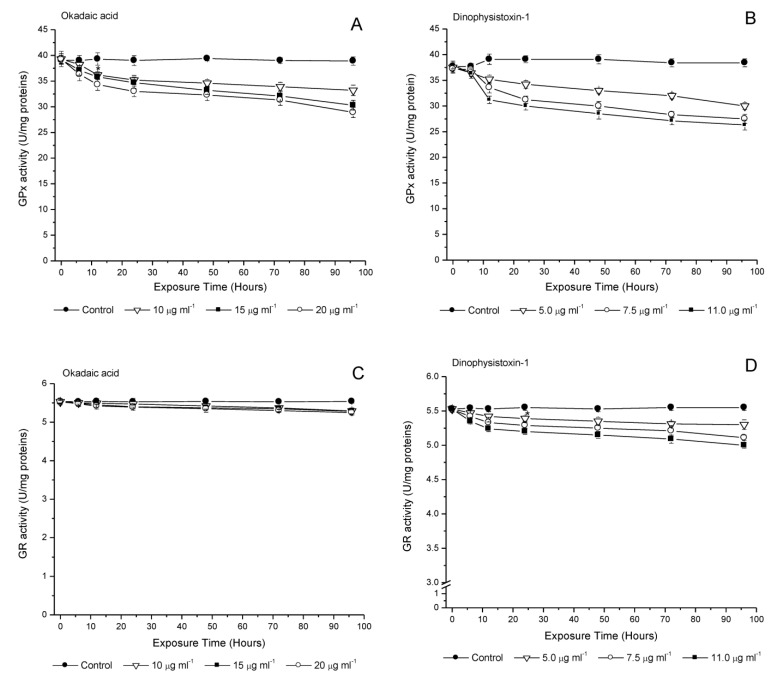
Glutathione peroxidase (GPx) (**A**,**B**) and glutathione reductase (GR) (**C**,**D**) activities in medaka larvae after exposure to okadaic acid (10; 15 or 20 μg/mL), and dinophysistoxins-1 (5.0; 7.5 or 11.0 μg/mL). Each of the OA-group toxin concentrations was compared with its respective control group. All values are shown as mean ± SEM (*n* = 3).

GPx enzyme activity decreased during incubation with OA or DTX-1 starting at 6 h (Figure 5A,B). Inhibition was dependent on the concentration used for both toxins during the 96 h incubation period. OA showed a slight difference in the decreased GPx enzyme activity in comparison to DTX-1, which showed a significant decrease at concentrations of 7.5 and 11.0 μg/mL between 6 h and 96 h of incubation (Figure 5B).

Finally, GR activity showed no significant changes at 96 h of incubation with OA at concentrations of 10, 15 and 20 μg/mL (*p* < 0.05) (Figure 5C). In contrast, DTX-1 incubation showed significant changes in GR activity from 12 h to 96 h of incubation (*p* < 0.05) at concentrations of 7.5 and 11.0 μg/mL. At a concentration of 5.0 μg/mL DTX-1, no changes in GR activity were detected (Figure 5D).

### 3.5. Effect of OA-Group Toxins on Protein Carbonylation and Lipid Peroxidation

**Figure 6:**
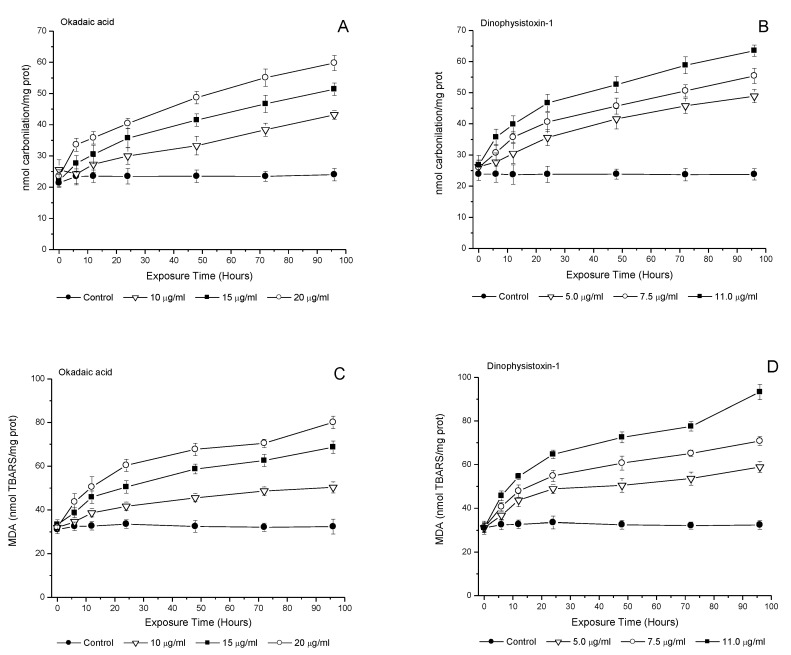
Carbonylation (**A**,**B**) and malondialdehyde (MDA) (**C**,**D**) contents in medaka larvae after exposure to okadaic acid (10; 15 or 20 μg/mL), and dinophysistoxins-1 (5.0; 7.5 or 11.0 μg/mL). Each of the OA-group toxin concentrations was compared with its respective control group. All values are shown as mean ± SEM (*n* = 3).

Protein carbonylation showed a significant increase after incubation with OA at concentrations of 15 and 20 μg/mL from 6 h up to 96 h of incubation (*p* < 0.05), with a marked trend from 24 h of incubation in all tested concentrations (10; 15 and 20 μg/mL) (Figure 6A). After incubation with DTX-1, protein carbonylation increased significantly relative to controls from 12 h at a concentration of 5.0 μg/mL, and from 6 h onwards at concentrations of 7.5 and 11.0 μg/mL, reaching a maximum variability with respect to the control at 96 h of incubation (*p* < 0.05) (Figure 6B).

Likewise, the levels of lipid peroxidation showed a significant increase in medaka larvae incubated at concentrations of 10, 15 and 20 μg/mL in OA, and 5.0, 7.5 and 11.0 μg/mL in DTX-1 (*p* < 0.05) versus the control incubation (Figure 6C,D). In addition, the incubation of medaka larvae with DTX-1 showed significantly higher levels of MDA from 6 h up to 96 h of incubation at concentrations of 5.0, 7.5 and 11.0 μg/mL (*p* < 0.05) (Figure 6D).

## 4. Discussion

HABs are considered natural environmental pollution events, as they produce different risks that are related to the production of chemical compounds and toxins in biological organisms [56,57,58]. In aquatic organisms, exposure to these pollutants can be analyzed by cellular, molecular, physiological and/or biochemical biomarkers [26,27]. Thus, the route and level of acute or chronic exposure will define the effects they may produce on biological organisms, such as changes in the community or population of specific aquatic organisms [59,60].

In this study, we show that acute exposure to OA-group toxin in medaka larvae (121–241 hpf) causes alterations in the antioxidant system, which in the early stages of development is associated with morphological alterations. The median lethal concentration of OA-group toxins in medaka larvae is specific; DTX-1 showed a higher toxic effect than OA [61,62]. Moreover, the morphological damage detected in medaka larvae at 121 hpf (tail injury and deformity, EC_50_) may coincide with that determined in digestive cell tissues (in vivo) [63,64]. OA has been described to exert a direct action on the cell cytoskeleton. It may induce microtubule destabilization, loss of stabilization of focal adhesions with consequent loss of cytoskeleton organization, and loss of barrier properties in different cell types [28].

Furthermore, the morphological damage produced by OA and DTX-1 in larvae at 121 hpf during 96 h of exposure to toxins evidences a critical point of toxin exposure in the development of medaka larval tissues. The most prevalent alterations in their development include severe pericardial edema, shortening of the anterior–posterior axis and developmental delay, which tends to decrease at lower concentrations of the toxin (<EC_50_). This damage could be related to the toxic equivalency factor (TEF) data shown for each toxin (OA: 1.0 TEF; DTX-1: 1.4 TEF) [62]. In addition to the time- and concentration-dependent morphological changes of the OA-group toxins, a significant decrease in the defense system involving antioxidant enzymes was detected [23,26]. The data showed a decrease in SOD and CAT levels from 48 h of exposure to OA (15 and 20 μg/mL) and from 12 h to DTX-1 (5.0; 7.5 and 11.0 μg/mL) in a concentration-dependent relationship. The decrease in the enzymatic activity of this first complex from 24 h of exposure correlates with the damage detected in the morphology of medaka larvae. This trend is maintained at up to 96 h of exposure, which could be explained by an inactivation of enzymatic proteins [65] and by a decrease in protein synthesis [66]. Likewise, at low concentrations of OA (10 μg/mL), no alteration in SOD activity was observed. As for DTX-1 exposure (5.0 μg/mL), a slight increase in SOD activity was observed, which could be explained by the activation of molecular mechanisms that allow for the synthesis of new SOD units, or activation of existing enzymes [67]. This suggests that at low concentrations of OA and DTX-1, in addition to the larval time-course (>180 hpf), the synthesis of antioxidant enzymes tended to increase.

It should be noted that the alterations detected in enzyme activities were always more evident when exposed to DTX-1, where there was a more significant decreased activity in SOD compared to OA. This could be related to the higher hydrophobicity of DTX-1 that favors interaction and permanence in medaka larvae tissues [26,68].

The detected decrease in CAT activity allowed us to determine that the level of oxidative stress induced by OA or DTX-1, in a toxin-dependent manner, reflects that a high level of ROS was not removed by CAT. In relation to DTX-1 exposure in zebrafish (*Danio rerio*), Figueroa et al. [26] showed that CAT decreased its activity due to the high levels of ROS produced by the toxins, which would cause direct damage to the enzyme. ROS, such as superoxide anion, reduces SOD activity, and at high concentrations of OA-group toxins, dual damage to the SOD–CAT complex can occur [24]. However, the results allow us to determine that at low concentrations of exposure to OA-group toxins, a trend to increase the SOD–CAT enzymatic activities is observed from 72 h, indicating that a response against toxins on ROS formation of the antioxidant system of medaka larvae occurs, but this increase in activity is not sufficient to decrease ROS levels, since approximately 14 days must elapse after exposure to xenobiotics for the recovery in the activity of this first oxidative barrier (SOD-CAT) [69].

In addition, GPx and GR showed a significant decrease in their activity when exposed to OA-group toxins at varying concentrations, evidencing a direct relationship with the low response of SOD and CAT. Previous studies have established that CAT and GPx act together in the uptake of ROS (hydrogen peroxide and hydroperoxides) [70,71]. Thus, low CAT activity makes it impossible to control the high levels of ROS produced by high concentrations of OA-group toxins (>7.5 μg/mL), directly affecting GPx and GR activity.

Based on the detected alterations of the antioxidant enzyme system, such as SOD and CAT, which act on hydrogen peroxide and superoxide anion, respectively, and GPx, which removes hydrogen peroxide and lipid hydroperoxides, it is established that at high concentrations of exposure to OA-group toxins, significant damage occurs in medaka larvae, leading to a significant imbalance between the activity of the antioxidant enzyme complex and the levels of ROS produced [26,72]. The data showed that DTX-1 induces greater damage in the medaka larvae due to a significant increase in TBARS levels, which is characterized by a 1.6-fold increase in LPO compared to OA. This could be explained by the fact that the higher toxicity of DTX-1 may cause a low response of the antioxidant enzyme system and an increase in LPO, leading to a more evident loss in the integrity of lipid membranes in medaka tissues in early developmental stages [15,16,73].

OA-group toxins are characterized by their ability to inhibit protein phosphatases 1 and 2A, so that acute exposure to the toxins can potentially produce significant biochemical alterations in aquatic organisms. With the inhibition of protein phosphatases (IC_50_ 0.1–10 nM), an increase in the phosphorylation of antioxidant enzymes subunits may occur and, as a consequence, alterations in the enzyme activities (e.g., alteration in the synthesis of SOD, CAT, GPx and GR enzymes), preventing an adequate response to xenobiotics or exposure to toxins [16,21,74]. It has been shown that reactive nitrogen species can also affect PP2A holoenzyme activity. Sripathi et al. [75] determined that nitric oxide signaling modulates PP2A activity through posttranslational modifications of the catalytic subunit, while Low et al. [76] established that peroxynitrite, which is formed through the association of the superoxide radical with nitric oxide, results in inactivation of PP2A.

The results obtained in this work lead us to propose that the morphological alterations detected in medaka larvae in the first hours of exposure may be the result of the synergistic effect produced by OA-group toxins (e.g., action on the cytoskeleton and inhibition of PP2A) and ROS (e.g., inhibition of antioxidant enzymes activity and increase of LPO), so that the combined effect causes oxidative damage. Therefore, EC_50_ concentrations in larvae < 217 hpf would produce an increase in morphological alterations. However, as larval development advances (hpf), a functional recovery of the enzymatic systems is expected given the increased organ development, allowing for an increase in the survival rate of larvae exposed to OA-group toxins [69], although it is possible to detect larval death at concentrations of 20 μg/mL of OA and 11 μg/mL of DTX-1 in the time range between 72 and 96 h of exposure (≈5%).

Moreover, the inhibition of oxidative enzymes and the increase in LPO are consistent with the results obtained in zebrafish (*Danio rerio*) exposed to 8.0 μg/mL and 6.0 μg/mL of OA and DTX-1, respectively [26]. Notwithstanding, according to the data obtained in this study, medaka larvae appear to be more resistant to oxidative stress caused by OA-group toxins by exerting specific effects on the SOD, CAT, GPx and GR enzymes at higher concentrations of OA or DTX-1. Thus, comparison of zebrafish and medaka data suggest that there are differential antioxidant responses between species. The interaction between toxin doses (OA or DTX-1), the duration of larval exposure to these toxins, and the abundance of the different toxic analogues (ratio of OA to DTX-1), is expected to greatly affect the oxidative responses determined in the different species [25,77]. However, it is clear that fish larvae in early life stages (<217 hpf) may be more susceptible to OA-group toxins when compared to juvenile and adult fish [78], so that low concentrations of OA-group toxins in the muscle tissue of juvenile and adult fish can be detected [79]. This could increase the risk of bioaccumulation and, consequently, the risk of cell damage, physiological alterations, behavioral changes and increased susceptibility to exposure to other toxins produced by HABs [80,81].

However, the results may contrast with those of other aquatic species. Studies in bivalves have determined that OA-group toxins are eliminated in a species-specific manner, which may be preceded and favored by biotransformations (C7 acylation), whereby conformational modifications of the toxins occur, modifying the ability to inhibit PP2A [15,74,82].

In summary, our results showed that acute doses of OA-group toxins (10, 15 and 20 μg/mL OA and 5.0, 7.5 and 11.0 μg/mL DTX-1) resulted in a decrease in the activity of SOD (≈25%), CAT (≈55%) and GPx and GR (≈35%). While the levels of MDA and carbonyl content increased significantly (≈50%) at the same concentrations of OA/DTX-1, this induced sustained morphological damage in the medaka larvae.

The increase in variables associated with climate change has led to an imbalance in the interaction of abiotic and biotic components in the sea, leading to the detection of a high incidence of HABs worldwide [5]. Climate variability in the South Pacific fjord zone has produced a severe imbalance from an oceanographic point of view (e.g., increased glacier melting, temperature variation, salinity and organic matter), leading to the detection of HABs associated with *Dinophysis* sp at different times of the year at levels > 118,000 cel/L [83]. Therefore, from an ecotoxicological point of view, exposure to OA-group toxins alters biomarker responses, producing a decrease in antioxidative activity, as well as an increase of biomarkers involved in lipid damage (LPO). Thus, the spatial and temporal variability of blooms in the southern fjords related to the production of OA-group toxins could affect the spawning periods and larval stages of the different endemic species present in the region.

**Figure 7:**
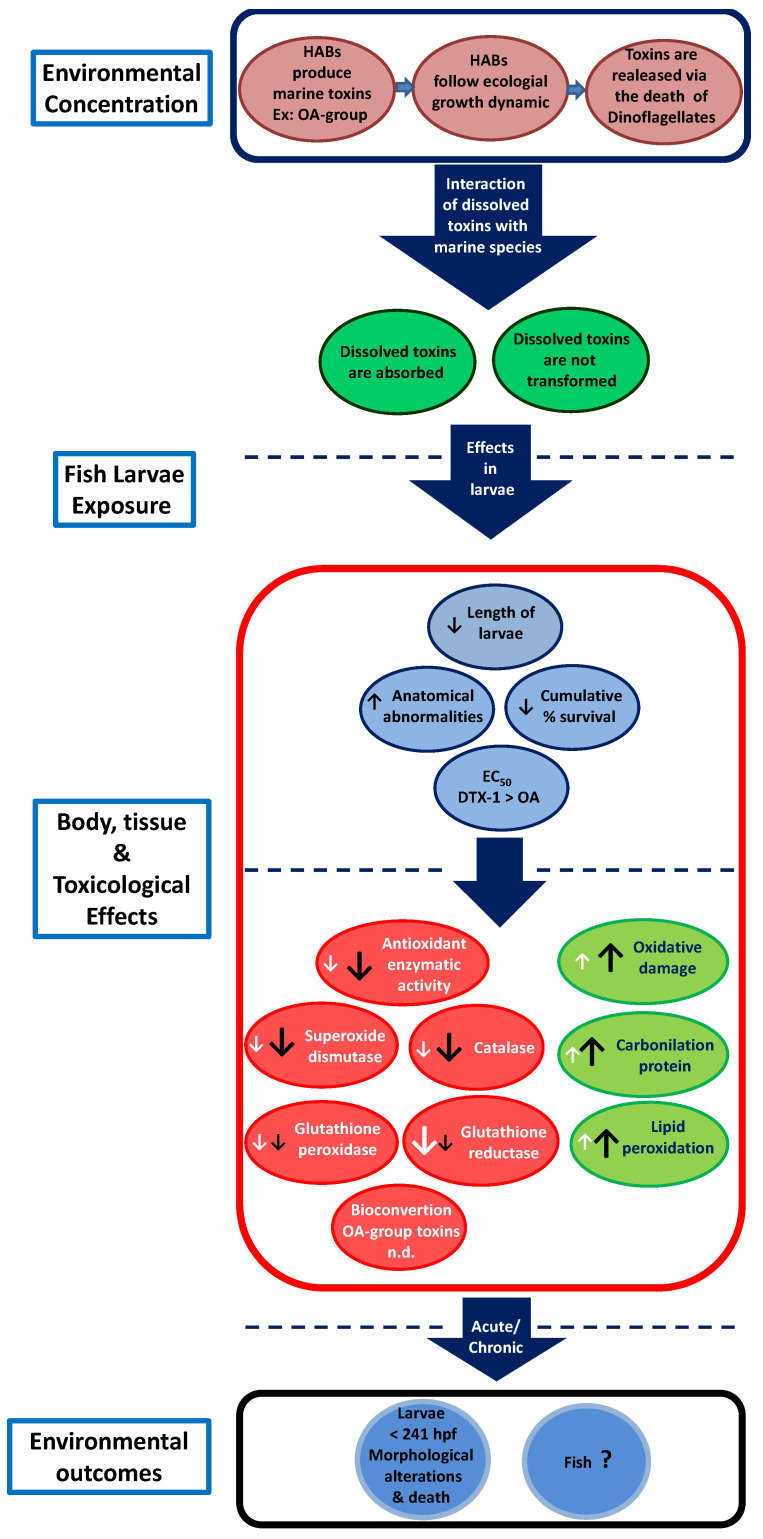
Conceptual diagram of how OA-group toxins produced by HABs, when released from dinoflagellates by cell lysis, can be absorbed by fish larvae producing malformations and alterations in their development (light blue circles), with a concomitant decrease in antioxidant enzyme activity (SOD, CAT. GPx and GR; red circles) and oxidative damage (carbonyl content and MDA, green circles). n.d. = not detected (black arrows indicate effect on zebrafish larvae and white arrows indicate effect on medaka larvae).

HABs associated with *Dinophysis acuta* cause the passive release of OA-group toxins, which induces a defensive response in fish, i.e., the activation of antioxidants (SOD-CAT/GPx-GR), whereas acute, single exposure in fjords can led to a significant decrease in antioxidant activities. The use of larval stages of zebrafish and medaka has allowed us to determine that exposure to OA-group toxins can alter biomarker responses, producing a decrease in antioxidative activity, as well as an increase of biomarkers involved in lipid damage (LPO). Thus, increased activity in the antioxidant system allows for a decrease in oxidative stress as larval development proceeds, since morphological alterations will be detected in early larval stages of development, with consequent larval mortality (Figure 7) [30,47,67].

## 5. Conclusions

In the present study, a significant level of toxicity was demonstrated in medaka larvae exposed to OA-group toxins (OA and DTX-1). The effects correlated with the concentrations of toxins to which the larvae were exposed, resulting in morphological alterations related to acute exposure (<EC_50_). These alterations were significant in larvae <180 hpf, and were directly linked to a decrease in the antioxidant enzyme system in a toxin-concentration dependent manner, over time. At high concentrations of OA (15 μg/mL) and DTX-1 (7.5 μg/mL), a decrease in SOD, CAT, GPx and GR enzyme activity was observed in ≈160 hpf larvae over 96 h of exposure. In contrast, at low toxin concentrations (10 μg/mL OA and 5.0 μg/mL DTX-1), an increase in SOD and CAT activity was observed at 48 h of exposure, where these levels failed to reduce oxidative damage. In addition, the high concentration of OA-group toxins reflects high levels of LPO from 12 h of toxin exposure. The results also demonstrate the higher toxicity of DTX-1 relative to OA, highlighting the severe environmental damage to early larval stages to which different endemic species may be exposed, considering that DTX-1 is the most prevalent analogue detected in blooms in the southern fjords of the South Pacific.

## Figures and Tables

**Table 1:** Mean frequency (%) of morphological abnormalities of medaka larvae exposed to OA-group toxins.

Abnormalities	Control	Exposed OA *	Exposed DTX-1 *
Edema	0	10	15
Cyclopia	0	5–10	10
Lethargy	0	40	40
Paresis	0	70	70
Touch responses	0	60	80
Craniofacial deformities	0	5	5–10
Pericardial edema	0	5	10–15
Spinal deformities	0	5–8	10
Reduced eye pigmentation	0	5	10
Developmental rate			
Shortening of the anterior-posterior axis	0	10–15	20–25
Developmental delay	0	30	40

* OA: Okadaic acid; DTX-1: Dinophysistoxin-1.

## Data Availability

Not applicable.

## Supplementary Materials

The following supporting information can be downloaded at: https://www.mdpi.com/article/10.3390/life13010015/s1, Figure S1. OA-group toxin exposure eliminated the response of larvae to a stimulus after being exposed to 10 and 15 μg/mL. Figure S2. Comparative photographs between 24 h control and treated (15 μg/mL OA group toxin) larvae of medaka fish (Cyclopia, Cy; shortening of the anterior-posterior axis, SP; and dorsal body curvature, DC).
