# Supplementary material for: Oxidative Stress Parameters and Morphological Changes in Japanese Medaka (Oryzias latipes) after Acute Exposure to OA-Group Toxins
Source: Life (Basel). 2022 Dec 21;13(1):15. doi: 10.3390/life13010015 (PMC9867479; doi:10.3390/life13010015)
Supplement: Supplementary file 1 [file life-13-00015-s001.zip › life-2085413-supplementary.pdf]

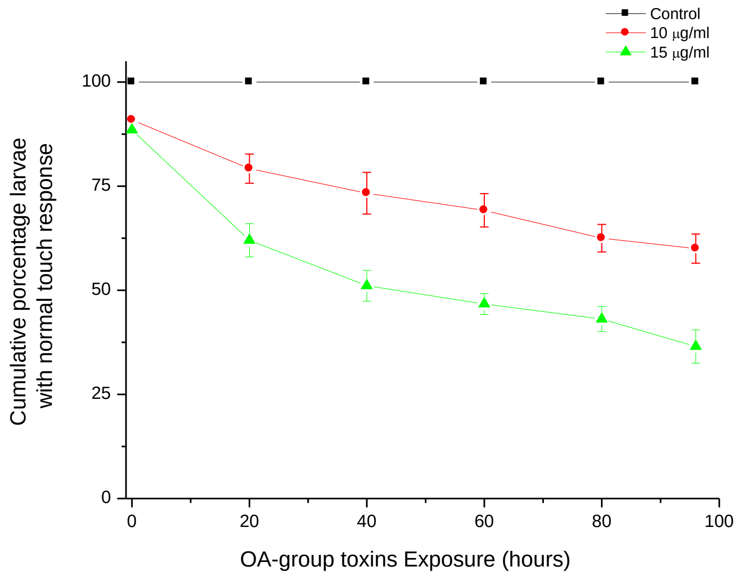

Figure S1. OA-group toxin exposure eliminated the response of larvae to a stimulus after being exposed to 10 and 15 µg/ml. All treatments consisted of three replicates containing five larvae each and are plotted as mean ± S.D.

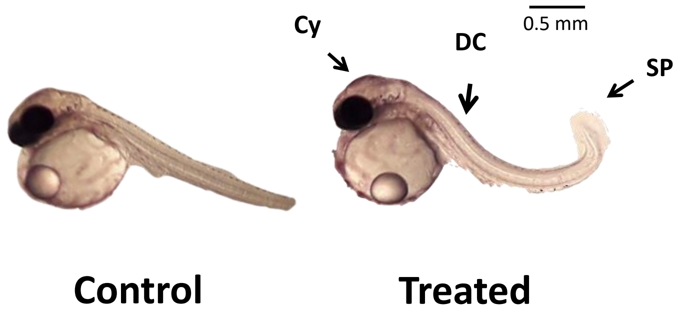

Figure S2. Comparative photographs between 24 h control and treated (15 µg/ml OA group toxin) larvae of medaka fish (Cyclopia, Cy; shortening of the anterior-posterior axis, SP; and dorsal body curvature, DC).
